# The complete mitochondrial genome of sponge *Halichondria okadai* (Demospongiae, Suberitida, Halichondriidae) from Korea water

**DOI:** 10.1080/23802359.2017.1407693

**Published:** 2017-11-27

**Authors:** Hana Kim, Hyung June Kim, Yun Hwan Jung, Cheol Yu, Yong Rock An, Donguk Han, Dong Won Kang

**Affiliations:** a Department of Taxonomy and Systematics, National Marine Biodiversity Institute of Korea Ecosystem, Seocheon-gun, Republic of Korea;; b Department of Biological Sciences, Inha University, Incheon, Republic of Korea

**Keywords:** Mitochondrial genome, *Halichondria okadai*, Halichondriidae, Demosponge

## Abstract

The mitogenome sequence of Sponge *Halichondria okadai* (Kadota, 1922) (Suberitida, Halichondriidae) was determined for the first time in this study. The circular genome is 20,722 bp in length, containing 14 protein coding genes (PCGs), two ribosomal RNAs (rRNAs), and 25 transfer RNAs (tRNAs). The nucleotide composition of mitogenome consists of 29.5% A, 14.2% C, 21.5% G, 34.7% T, showing a high content of A + T similar to the other Suberitid sponges. These results will be useful for inferring the phylogenetic relationships among the members of family Halichondriidae within the Suberitids.

*Halichondria okadai* is a thin encrusting species belonging to family Halichondriidae and inhabits in the intertidal zone. Sponges of the genus *Halichondria* Fleming, 1828 are presently recognized as 93 valid species in the world ocean (van Soest et al. 2017) and they are distributed over all regions and habitats (Hooper and van Soest 2002). Twelve species of the genus *Halicondria* have been reported from Korean waters (NIBR 2012). It is known that the drug Eribulin extracted from *H. okadai* has the effect of extending the life span of women with breast cancer (Cortes et al. 2011; Ohno et al. 2011). Until now, the only two species (*Halichondria* sp., *Hymeniacidon sinapium*) of Halichondriid have been reported for their complete mitochondrial genomes (Jun et al. 2015; Wang et al. 2016). Here we report the complete mitochondrial genome (mitogenome) of *H. okadai* for the first time.

Specimens of *H. okadai* were collected from intertidal zone of Jeju Island of Korea. The voucher specimens were deposited in National Marine Biodiversity Institute of Korea (MABIK IV00163014-00163019). The genomic DNA was isolated from the tissue and the mitogenome sequences were analysed by application of Illumina Hiseq2000 sequencing platform (Macrogen, Seoul, Korea). The sequences were assembled and annotated in comparison with the previously reported mitogenome sequences of Halichondriid species (Jun et al. 2015; Wang et al. 2016) using Geneious v9.1.8 (Kearse et al. 2012). Phylogenetic tree was constructed using MEGA6 (Tamura et al. 2013).

The complete mitogenome of *Halichondria okadai* (GeneBank accession number MG267395) is 20,722 bp in length, containing 14 protein coding genes (PCGs), two ribosomal RNAs (rRNAs), and 25 transfer RNAs (tRNAs). The overall nucleotide base composition of *H. okadai* is 29.5% A, 14.2% C, 21.5% G, 34.7% T, showing a high A + T bias (64.2%) similar to the other Halichondriid sponges. All PCGs use the typical ATG as the start codon. Ten PCGs (*atp9, cytb, cox3, atp6, cox2, nad1, cox1, nad4l, nad6,* and *nad4*) use TAA as the stop codon while four (*atp8, nad5, nad2, nad3*) genes have TAG. The lengths of tRNA genes range from 71 to 80 bp and all tRNAs have the typical clover leaf structure. The sizes of 12S and 16S rRNAs are 1364 bp and 2834 bp, respectively.

The taxonomic position of *H. okadai* analysed by molecular phylogenetic tree of combined 14 PCGs sequences using the neighbour-joining (NJ) method with the K2P model in MEGA 6. *H. okadai* were clustered together as *Halichondria* sp. (KX244759), previously announced from GenBank. Their cluster is strongly supported by bootstrap values of 100% (Figure 1). The similarity of mitogenome between *H. okadai* and *Halichondria* sp. is 99.6%, they were same species. To conclude, we confirmed that *Halichondria* sp. reported by Wang et al. (2016) was *H. okadai*. In this study, we determined the complete mitogenome of sponge *H. okadai*, and it will provide useful information for understanding of evolutionary history and phylogeny of the genus *Halichondria* in relation to the other genera within family Halichondriidae.

**Figure 1.**
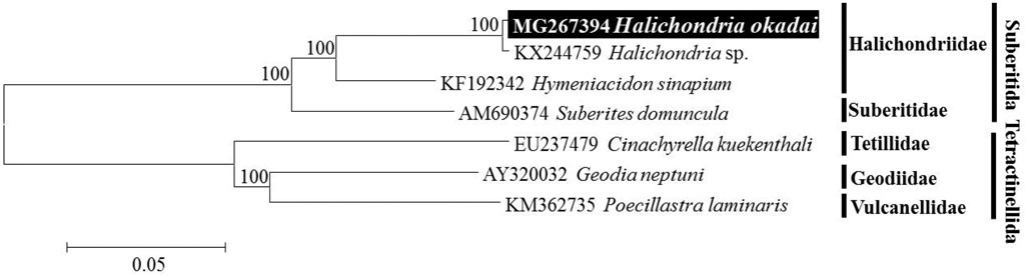
Neighbour-joining (NJ) tree based on the mitogenome sequences of three Halichondriid species including *Halichondria okadai* with two other related species in Suberitida. Three species (*Cinachyrella kuekenthali*, *Geodia neptuni*, *Poecillastra laminaris*) derived from Tetractinellida was used as outgroup for tree rooting. Numbers above the branches indicate NJ bootstrap values from 1000 replications.
